# Characteristics Associated with Failure of Obstetric Fistula Surgery

**DOI:** 10.1007/s00192-026-06596-6

**Published:** 2026-04-14

**Authors:** Hannah Chapman, Amadou Issa Abdou, Chelsea Porter, Guihua Zhai, Isuzu Meyer, Itengre’ Ouedraogo, Yakoubou Sanoussi, Abdoulaye Idrissa, Holly E. Richter

**Affiliations:** 1 Department of Obstetrics and Gynecology, University of Alabama at Birmingham, 1700 6Th Av South, Suite 10382, Birmingham, AL 35233 USA; 2 Department of Obstetrics and Gynecology, Dan Dicko Dankoulodo University of Maradi, Maradi, Niger; 3 Danja Fistula Center, Danja, Niger; 4 Center for Clinical and Translation Science, University of Alabama at Birmingham, Birmingham, USA; 5 ARENA, Centre Medical Renaissance, Ouagadougou, Burkina Faso; 6 Galmi Hospital, Galmi, Niger; 7 Reseau d’Eradication de La Fistule, Niamey, Niger

**Keywords:** Obstetric fistula, Risk factors for surgical failure, Vesicovaginal fistula, Vesicovaginal fistula repair

## Abstract

**Introduction and Hypothesis:**

Quality of life can significantly improve following successful surgical repair of obstetric fistula; however, these surgeries may be technically challenging. There are limited consensus data regarding optimal fistula closure techniques and perioperative management. The objective of this study was to identify clinical and anatomical characteristics associated with failure of obstetric vesicovaginal/urethrovaginal fistulas (VVaF/UVaF) closure.

**Materials and Methods:**

In this retrospective cohort study, medical records of patients following VVaF/UVaF surgery at a single fistula center in Niger between 2022 and 2024 were identified. Clinical and demographic data were collected. Successful fistula closure was defined as water-tight visually assessed closure of fistula at discharge from the fistula center. Multivariable logistic regression was used to identify independent factors associated with closure failure.

**Results:**

Of 125 women, 28 (22.4%) had failure of fistula closure. Mean age (SD) was 31.2 (9.78) years and median (interquartile range [IQR] parity 2 (0–4), with no difference between groups (all *p* > 0.05). Multivariable logistic regression adjusting for age resulted in an aOR (95% CI) for the number of previous surgeries of 1.37 (1.06, 1.75), and Goh’s fibrosis level 2 vs 1 of 2.70 (1.04, 7.14) and 3 vs 1, 4.55, (1.04, 20.0).

**Conclusions:**

In this population of women undergoing obstetric VVaF/UVaF repair, patients having undergone more surgeries and those with greater tissue fibrosis had greater odds of failed fistula closure. This emphasizes the importance of the initial fistula repair surgery as the best chance for fistula closure in these patients sustaining life-changing pelvic floor injury.

## Introduction

Obstetric genitourinary fistulas are a serious complication of obstructed childbirth and defined as an abnormal opening between the vagina and urinary system, resulting in continuous urine leakage through the vagina [1]. In Sub-Saharan Africa, obstetric fistulas are estimated to occur in 1 to 3 out of every 1000 deliveries [2]. The World Health Organization (WHO) estimates globally that there are 30,000 to 130,000 new obstetric fistula cases in Africa annually, with the majority in Sub-Saharan Africa alone [3].

Obstetric fistulas impact every aspect of a woman’s quality of life. They are associated with a significant impact on daily activities, economic consequences, and physical and psychological wellbeing. Household relationships, community participation and socialization can be particularly impacted owing to the stigma and isolation associated with the diagnosis. Severe depression and other mental health co-morbidities are common among patients affected by genitourinary fistula [4–6].

Quality of life can significantly improve following successful surgical repair of obstetric vesicovaginal/urethrovaginal (VVaF/UVaF) fistulas; however, these surgeries may be technically challenging and there are limited consensus data regarding optimal fistula closure techniques and perioperative management [7, 8]. Even after successful repair, there is also the risk of ongoing urethral incompetence and urinary incontinence, which can be challenging to treat and refractory to current evidence-based surgical approaches [9].

The objective of this study was to identify clinical and anatomical characteristics associated with failure of obstetric VVaF/UVaF closure linked to surgery and perioperative care.

## Materials and Methods

This was a retrospective cohort study conducted at a single fistula center in Niger. The institutional review board at the University of Alabama at Birmingham approved this study. Local institutional approval was also obtained from the Danja Fistula Center Clinical Oversight and Ethics Study Review Committee with waived patient consent. Medical records of surgical patients following obstetric VVaF/UVaF surgery between 2022 and 2024 were identified by chart review and all data were kept de-identified. Clinical and demographic data were collected using a secure electronic standardized chart abstraction form. Failure of VVF closure was defined as fistula visualized with dyed bladder fill on examination prior to discharge from the fistula center by the operating surgeon. Patients remained at the fistula center until discharge at 6–12 weeks postoperatively, which is standard of care in this fistula center. Controls were women with successful fistula closure, defined as water-tight visually assessed on examination with dyed bladder fill, prior to discharge from the fistula center.

Patient demographic and clinical information was collected, including age, gravidity, parity, hours in labor, fistula from pregnancy, mode of delivery, neonatal mortality, duration of fistula by self-reported date of injury, previous fistula surgery, number of fistula surgeries, surgical approach, estimated blood loss, starting hemoglobin (Hgb), residual urinary incontinence, postoperative urinary retention with postvoid residual

 > 150 ml, duration of Foley catheter use postoperatively, and Gohs classification, including distance from the external urethral meatus, size of the fistula, and severity of fibrosis [10]. Residual urinary incontinence was documented as any leaking per urethra, even in those patients with failure of obstetric VVaF/UVaF closure.

Study data were collected and managed using secure electronic data tools at the University of Alabama at Birmingham. Data were compared between successful and unsuccessful fistula closure groups using Student’s *t* tests or Wilcoxon’s rank sum tests for continuous variables, and Chi-squared test for categorical variables, as appropriate. All data were presented as mean (SD) or median for continuous variables and number (%) for categorical variables. Baseline characteristics in bivariate analysis with *p* values ≤ 0.1 and deemed clinically significant a priori were assessed for association with unsuccessful fistula closure by logistic regression while controlling for age. The final models had two variables, each of the selected variables and age. Collinearity was also assessed between each variable and age using variance inflation factor (VIF). Statistical analysis was performed using SAS software version 9.4 (Cary, NC, USA). Statistical significance was defined at the 5% level.

## Results

Between 2022 and 2024, a total of 125 patients underwent obstetric vesicovaginal/urethrovaginal fistulas repair. Chart review of postoperative data revealed *n* = 28 with failure of fistula closure, defined as persistent fistula visualized on examination with dyed bladder fill prior to discharge from the fistula center 6–12 weeks after surgery. Clinical and demographic characteristics of the subjects with fistula closure failure were compared with the 97 subjects with fistula closure success (Table 1). Overall, subjects had a mean (SD) age of 31.2 years (9.8) and median (IQR) parity 2 (0–4). Subjects with obstetric fistula surgery failure had a longer duration of having a fistula, a greater number of surgeries, and greater tissue fibrosis. They were more likely to have undergone obstetric fistula surgery, and the location was more likely to be closer to the external urethral meatus (all *p* < 0.05, Table 1). No other significant differences were noted between the two groups.

**Table 1:** Clinical and demographic characteristics of women with unsuccessful versus successful fistula closure

Characteristics	Total	Unsuccessful closure (*n* = 28)	Successful closure (*n* = 97)	*p* value
Age, years, mean (SD)	31.15 (9.78)	33.96 (7.61)	30.34 (10.21)	0.08
Gravida, mean (SD)	4.53 (3.31)	4.00 (3.20)	4.68 (3.35)	0.34
Parity, median (IQR)	1 (0, 4)	1 (0, 3)	2 (0, 4)	0.20
Fistula from para, median (IQR) ^a^	4 (1, 6)	3 (1, 5)	4 (1, 6)	0.20
Hours in labor, mean (SD)	56.90 (36.44)	55.38 (38.86)	57.33 (35.92)	0.81
Mode of delivery, *n* (%)				0.38
Vaginal	47	14 (50.00)	33 (35.48)	
Cesarean	57	11 (39.29)	46 (49.46)	
Operative	17	3 (10.71)	14 (15.05)	
Neonatal mortality, *n* (%)				0.74
Yes	107	24 (85.71)	83 (89.25)	
No	14	4 (14.29)	10 (10.75)	
Duration of fistula, months	55.22 (80.39)	90.50 (107.39)	45.04 (68.09)	0.04
surgery, *n* (%)				0.001
Yes	51	19 (67.86)	32 (32.99)	
No	74	9 (32.14)	65 (67.01)	
Number of previous surgeries, median (IQR)	0 (0, 2)	2 (0, 3)	0 (0, 1)	0.01
Previous surgery at DFC, *n* (%)				0.89
Yes	79	16 (57.14)	63 (64.95)	
No	46	12 (42.86)	34 (35.05)	
Surgery, *n* (%)				0.19
Vaginal VVF repair	114	24 (85.71)	90 (92.78)	
Abdominal VVF repair	6	3 (10.71)	3 (3.09)	
Vaginal VVF repair with sling	5	1 (3.57)	4 (4.12)	
Estimated blood loss, mean (SD)	163 (177.62)	155.36 (149.90)	165.21 (185.50)	0.79
Starting Hgb, mean (SD)	12.28 (1.46)	11.88 (1.30)	12.39 (1.50)	0.11
Residual UI, *n* (%)				0.20
Yes	28	9 (32.14)	19 (19.59)	
No	97	19 (67.86)	78 (80.41)	
Postoperative urinary retention, *n* (%)				0.20
Yes	9	0 (0)	9 (9.28)	
No	116	28 (100)	88 (90.72)	
Foley duration, days, median (IQR)	17.53 (5.00)	19.00 (6.10)	17.10 (4.58)	0.14
Gohs location—distance from external urethral meatus, *n* (%)				0.01
Type 1 > 3.5 cm	71	10 (35.71)	61 (62.89)	
Type 2 2.5 to 3.5 cm	18	7 (25.00)	11 (11.34)	
Type 3 1.5 to < 2.5 cm	28	6 (21.43)	22 (22.68)	
Type 4 < 1.5 cm	8	5 (17.86)	3 (3.09)	
Gohs size, *n* (%)				0.29
A < 1.5 cm	87	17 (60.71)	70 (72.16)	
B 1.5 to 3 cm	22	5 (17.86)	17 (17.53)	
C > 3 cm	16	6 (21.43)	10 (10.31)	
Gohs fibrosis, *n* (%)				0.04
1 none or mild	61	8 (28.57)	53 (54.64)	
2 moderate or severe	54	16 (57.14)	38 (39.18)	
3 ureteric/circumferential	10	4 (14.29)	6 (6.19)	

*Danja Fistula Center*, *Hgb* hemoglobin, *IQR* interquartile range, *SD* standard deviation, *UI* urinary incontinence, *VVF* vesicovaginal fistula

Although previous surgery was significant, we only assessed the number of previous surgeries so that risk could be defined per additional prior repair. Additionally, starting Hgb was assessed with *p* = 0.11. Variation inflation factors in all final models were close to 1, indicating no collinearity. Multivariable logistic regression adjusting for age resulted in an aOR (95% CI) for the characteristic of number of previous surgeries of 1.37 (1.06, 1.75), where one more surgery increased the odds of VVaF/UVaF surgery failure by 37% per additional prior repair. The odds ratios of fistula closure failure for Gohs fibrosis level 2 vs 1 and level 3 vs 1 were 2.7 (1.04, 7.14) and 4.55 (1.04, 20.0) respectively. The odds of fistula closure failure for patients with type 2 Gohs fibrosis was 2.7 times the odds of failure in patients with type 1 fibrosis, and the odds of fistula closure failure for type 3 fibrosis was 4.55 times the odds of fistula closure failure in subjects with type 1 Gohs fibrosis (Table 2).

**Table 2:** Logistic regression assessing the association between variables and fistula closure failure

^a^Characteristics	Odds ratio	CI (95%)	*p* value
Duration of fistula, months			0.07
> 6 months	1.03	(0.99, 1.06)	
Number of previous surgeries	1.37	(1.06, 1.75)	0.01
Gohs location: distance from external urethral meatus			0.06
3 + 4 vs 1 + 2	2.48	(0.96, 6.43)	
Gohs fibrosis			0.05
2 vs 1	2.70	(1.04, 7.14)	
3 vs 1	4.55	(1.04, 20.0)	
Starting Hgb	0.80	(0.59, 1.09)	0.06

*Hgb* hemoglobin

^a^Controlling for age

## Discussion

In women undergoing obstetric VVaF/UVaF repair at a fistula center in Niger, patients having undergone more surgeries and those with greater tissue fibrosis had greater odds of failure of fistula closure. This emphasizes the importance of the initial fistula repair surgery as the best chance for fistula closure in these patients, as multiple surgeries can increase the risk for fibrosis.

Despite the known importance of the initial fistula repair surgery, there is limited evidence behind surgical techniques and perioperative care [8]. Surgical techniques are well described but are entirely based on expert opinion. A vaginal approach is generally preferred by most surgeons, but suture material and number of layers used for closure have not been studied in a systematic way [11]. Tissue and muscle flaps have also recently been used for vaginal reconstruction to improve closure rates and postoperative continence but have not been studied in a systematic way to ensure proper patient selection either [12, 13].

The finding that degree of scarring (as measured by Goh’s fibrosis level) was associated with failure of fistula repair is consistent with findings in other single countries including Ethiopia, Uganda, and the Democratic Republic of Congo and in a study summarizing data from five countries including Bangladesh, Guinea, Niger, Nigeria, and Uganda [14–17]. Number of previous repairs has also been associated with failure of fistula closure, where in one study, compared with primary closure, odds of failure of fistula closure was almost five times greater than for three or more repairs (OR 4.7; 95% CI 2.2, 10.0). In a recent systematic review and meta-analysis, pooled prevalence of obstetric fistula repair failure in Sub-Saharan Africa was 24.92% (95% CI 20.34–29.50); characteristics associated with failure of fistula repair included previous fistula repair surgery (OR 2.70; 95% CI 1.94, 3.45), along with total urethral damage, fistula size, and duration of labor [18]. These studies demonstrated an even stronger association between prior fistula repair and failure of surgery. In the current study, Gohs fibrosis had a higher impact on fistula closure failure than number of previous surgeries and duration of fistula (Table 2). Fibrosis as opposed to prior surgery may be more of a cause and marker of fistula complexity, and may correlate with location, size, and urethral involvement.

Urinary leakage post-obstetric fistula repair is another condition that impacts patients following surgery. Forces exerted on the lower urinary tract during obstructed labor, resulting in scarring, muscle and nerve injury, lead to devitalized tissue, and a potential impact on components of the continence mechanism. This has ongoing health impacts even after “successful” fistula closure and treatment can be challenging, as patients in these circumstances can be refractory to current evidence-based approaches. This is an important area for future research.

The major strength of this study was the large cohort of patients over a 3-year span at a single, remote fistula center managed in a standardized fashion by the same International Federation of Gynaecology and Obstetrics-certified surgeon. Preoperative evaluation, surgical technique, and postoperative care have been standardized at this center. Although only at a single institution, this reliable database allowed us to maximize the amount of information that could be collected from retrospective chart review. This database also allowed the assessment of surgical outcomes and perioperative considerations.

The limitations of this study included the retrospective design, which limited information that could be abstracted from the medical record. For instance, the surgical approach and Gohs staging including location, size, and fibrosis were consistently documented, but rationale for the approach was not standardized and was based on surgeon preference. The retrospective nature also limits the ability to infer causation, only association. The 6- to 12-week outcome range at discharge could have influenced detection of fistula closure failure in the event of late breakdown. Additionally, there was potential measurement bias, as the primary surgeon assessed the outcome at discharge.

Further research is needed to improve the treatment outcomes of obstetric VVaF/UVaF repair. Future research should seek an increased understanding of these factors associated with successful obstetrics-related fistula closure and perioperative care. Surgical technique and concomitant procedures to improve primary closure should be systematically studied to provide the best chance for fistula closure in these patients sustaining life-changing pelvic-floor injury.

## Data Availability

The data that support the findings of this study are available from the corresponding author upon reasonable request.
